# Ultrasonographic Assessment of Teat Structures in Healthy Lactating Jennies: A Pilot Study Establishing Reference Values for Clinical Application

**DOI:** 10.3390/vetsci12121123

**Published:** 2025-11-26

**Authors:** Lucrezia Accorroni, Andrea Marchegiani, Marilena Bazzano, Andrea Spaterna, Fulvio Laus

**Affiliations:** School of Biosciences and Veterinary Medicine, University of Camerino, Via Circonvallazione 93/95, 62024 Matelica, Italyfulvio.laus@unicam.it (F.L.)

**Keywords:** mammary gland, diagnostic imaging, milk yield, dairy donkey, equid

## Abstract

In recent years, donkey milk has gained growing interest for its nutritional and therapeutic properties, stimulating both research and commercial interest. Monitoring udder health is essential to reduce production losses and ensure animal welfare. Despite its importance, information about the ultrasonographic anatomy of teat structures in lactating jennies is limited, and normal reference values are not well established. This study aimed to describe the ultrasonographic appearance of the teat and provide reference measurements in healthy lactating standard dairy jennies. Twenty-eight subjects were examined using a 13 MHz linear transducer, and longitudinal and transverse scans were performed to assess teat canal length, teat canal diameter, and cranial and caudal teat wall thickness. All measurements were repeatable and showed high bilateral symmetry. Teat canal diameter was positively correlated with the month of lactation (*p* < 0.05), whereas no significant associations were found with age or body weight. These findings establish normative ultrasonographic parameters for teat structures in jennies and highlight the progressive adaptation of the teat canal during lactation. Standardized measurements can support early detection of mammary gland pathologies, guide preventive management of mastitis, and improve udder health monitoring in donkey dairy farming.

## Simple Summary

Donkey milk is becoming increasingly popular for its nutritional and therapeutic value, but keeping animals healthy is essential for ensuring good milk quality and welfare. The udder and its teats play a key role in milk production and protection against infections such as mastitis. However, limited information is available on their normal ultrasonographic appearance. This study used ultrasound to describe the internal structure of healthy, lactating donkeys and to measure key features such as the length and diameter of the teat canal and the thickness of the teat wall. Results showed that all teats had a similar structure on both sides of the udder and that the teat canal became slightly wider as lactation progressed. These measurements provide normal reference values that veterinarians can use to monitor udder health. Establishing these standards helps improve diagnosis, prevent mastitis, and support animal welfare and milk quality in donkey dairy farming.

## 1. Introduction

In recent years, equid milk obtained from jennies (*Equus asinus*) has attracted increasing attention due to its therapeutic properties, with applications in both the dietary and cosmetic fields [1]. Donkey milk contains less total protein and casein than bovine milk, but higher levels of whey proteins and mono- and polyunsaturated fatty acids [2,3,4,5,6]. Its close similarity to human milk in terms of lactose content, protein and amino acid composition, together with its hypoallergenic nature, makes donkey milk an excellent substitute in the dietary management of Cow Milk Protein Allergy (CMPA) in hypersensitive individuals, infants and elderly people [7,8,9,10,11].

In addition, donkey milk exhibits antimicrobial, antioxidant, anti-inflammatory and immunomodulatory effects, with potential applications in tumor suppression, diabetes management and dermatological therapy [8,12,13,14,15].

Mastitis is one of the most common and economically significant mammary gland diseases, typically arising when pathogens enter through the teat canal [16,17]. In equines, its incidence is lower than in ruminants, mainly due to the smaller size and storage capacity of the udder and its higher distance from the ground [18]. Nevertheless, mastitis can have severe consequences, including fibrosis, abortion, and transmission of the infectious agent to the nursing foal [18].

Early diagnosis of mammary gland disorders is essential to prevent economic losses, improve animal welfare and ensure hygienic milk production [16,17]. Investigating the structure and function of the mammary gland is therefore important, as it allows the early detection of pathological processes and supports their prompt treatment [16].

Ultrasonography is a rapid, safe, and non-invasive diagnostic technique widely used in dairy animals to assess mammary gland health [19,20,21,22,23]. Its application to teat structures is particularly relevant, as it allows the detection of alterations associated with mastitis as well as anatomical features that may predispose animals to the development of this pathology [17,22,24].

The donkey’s udder, similarly to that of the mare, sheep and goat, consists of a single pair of inguinal mammary glands, each bearing one teat [25]. However, in equids, each teat is typically drained by two main ductal systems, each ending in a separate orifice, in contrast to the single-duct arrangement of ruminants [25]. The glandular portion of each half is further divided by fibroelastic capsules into cranial and caudal lobes. Each of the two teats serves a cranial and caudal lobe of mammary tissue [26]. Nevertheless, it has been demonstrated that in mares it is sometimes possible for the teat to have only a single teat canal with one glandular complex [27].

Studies on the inner surface of the teat have revealed the presence of ring-like mucosal folds, located proximal to the opening of the teat canal [27]. These folds may function as defense structures, impeding the entry of bacteria and other pathogens [26]. Although it remains uncertain whether they can be considered an analog of the Fürstenberg rosette described in cattle—which is located at the junction between the pars papillaris of the sinus lactiferus and the teat canal—histological and cytological analyses indicate that the folds are composed of superficial epithelium and underlying connective tissue, with keratinized cell clusters contributing to mechanical and biological defense [27].

Current literature on ultrasonographic evaluation of teat structures in equids is limited [28,29,30] and mostly restricted to early lactation stages, with scarce information on examination protocols. The present study aimed to fill these gaps by assessing teat morphology across different months of lactation, providing more detailed reference measurements and evaluating the feasibility of ultrasonography as a reliable diagnostic method applicable under field conditions.

Therefore, the present observational, cross-sectional, and descriptive pilot study aimed to provide a detailed ultrasonographic description of teat structures in healthy lactating standard dairy jennies and to obtain preliminary reference data useful for clinical evaluation. An additional aim was to assess the feasibility of performing ultrasonographic examinations under real field conditions. These data may also guide future research involving larger, breed-balanced populations with a sufficient number of subjects in each age and lactation group.

## 2. Materials and Methods

### 2.1. Animals

**Figure 1:**
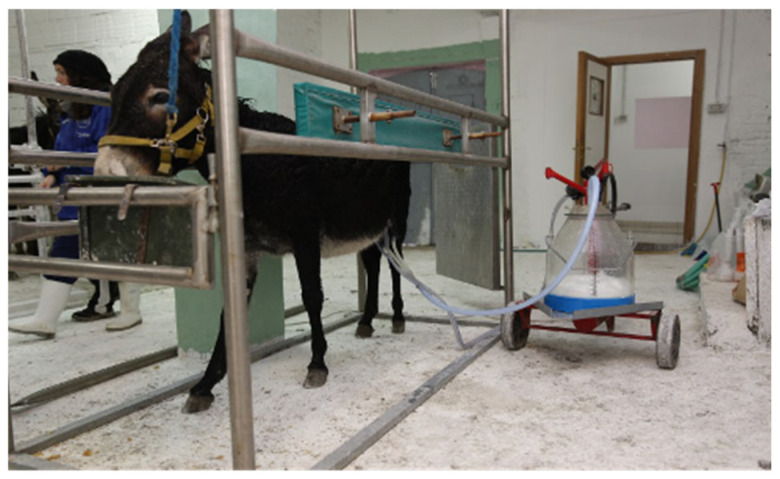
Mechanical milking of a donkey during routine farm procedures.

Twenty-eight adult mixed-breed standard dairy jennies (mean age 7.4 ± 3.1 years, range 2–13 years; age distribution: 2yo (*n =* 2), 3yo (*n =* 2), 4yo (*n =* 2), 5yo (*n =* 3), 6yo (*n =* 2), 7yo (*n =* 1), 8yo (*n =* 4), 9yo (*n =* 4), 10yo (*n =* 4), 11yo (*n =* 1), 12yo (*n =* 2), 13yo (*n =* 1); mean body weight 233.1 ± 58.7 kg) housed in the same semi-intensive farm and milked twice daily—in the morning between 06:00 am and 08:30 am and in the evening between 05:00 pm and 07:30 pm—were enrolled in the study. As a routine, foals were separated from their mothers two hours before milking. During the rest of the day, foals normally remained with their mothers and were free to suckle. Mechanical milking was performed using a portable milking machine for small ruminants with a vacuum between 38 and 42 kPa and a pulsation frequency of approximately 60 cycles/minute (Figure 1).

All lactating jennies from this farm were included, as they were easily manageable without the need for sedation. All included donkeys were lactating, some of which were examined ultrasonographically during the first (*n =* 1), third (*n =* 2), fifth (*n =* 5), sixth (*n =* 3), seventh (*n =* 6) or eighth (*n =* 11) month of lactation. Ultrasonographic examinations were all performed in the afternoon after the second session of milking,

A physical examination of each animal was performed to assess the health status together with udder evaluation through inspection, palpation and milk somatic cells count (SCC). The presence of any systemic alterations [31], clinical abnormality of mammary gland [18] or a SCC higher than 50,000/mL [32,33,34] was considered a reason for exclusion from the study.

### 2.2. Ultrasonographic Examination and Measurements

Ultrasonographic examinations were performed using a 13 MHz linear transducer (MyLab™One, Esaote, Genoa, Italy). During the examination, jennies were gently restrained in a standing position and the entire procedure lasted approximately 15–20 min per animal.

For the examination of the udder parenchyma, the transducer was applied directly to the skin after cleaning the surface with 90% isopropyl alcohol to remove debris and residual fat, and applying ultrasonographic gel to the probe to ensure optimal acoustic coupling.

For the examination of the teats, longitudinal and transverse scans were recorded with the probe cranial to the teat. The longitudinal scan was obtained by placing the linear probe parallel to the teat, while the transverse scan was obtained by rotating the probe 90° in order to clearly visualize the papillary orifice, teat canals, teat cisterns and teat wall. Ultrasound of the teats was performed with the “water bath” technique, by immersing the teat in a cup filled with warm water, with the probe placed on the outer surface of the cup.

Teat canal length (TCL), teat canal diameter (TCD), and cranial (CrTWT) and caudal (CaTWT) teat wall thickness were measured in both the right and left mammary glands of each jenny. All measurements were obtained by the same examiner and repeated on three consecutively created images; the mean value was subsequently used for statistical analysis.

**Figure 3:**
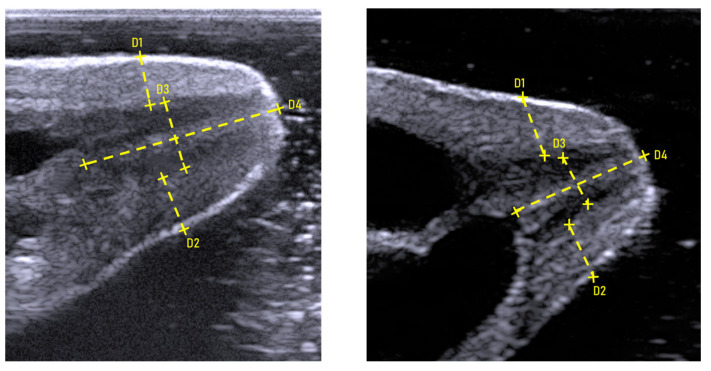
Longitudinal ultrasonographic image of a lactating jenny’s teat showing measurements of the teat cranial (D1) and caudal (D2) wall thickness, teat canal diameter (D3) and teat canal length (D4).

Teat canal length was measured from the visible proximal end of the teat canal to the teat orifice at the level of the septum dividing the two ductal systems. Teat canal diameter was measured between the hyperechoic inner borders of the teat canal wall at the midpoint between the teat orifice and the beginning of the teat cistern. This measurement includes both teat canals of a single teat, extending from the cranial border of the cranial teat canal to the caudal border of the caudal teat canal (Figure 3). Cranial and caudal teat wall thickness was measured from the outer hyperechoic epidermal layer to the inner mucosal layer at the mid-height of the teat canal.

### 2.3. Statistical Analysis

Statistical analysis has been performed using the software “R” (Version R 4.1.0, R Core Team, 2021). Repeatability has been determined according to the Bland and Altman plot. Descriptive statistics have been reported (Mean (SD), 95% IC, Percentile, Min, Max). First, data were checked using Shapiro–Wilk test for normality. The relationships between age and body weight with all measurements were assessed by Pearson correlation coefficient. To calculate the p-value of the Pearson correlation coefficient, the cor.test function in “R” has been used. The function uses the value of the correlation coefficient (r) and the number of observations (n) to determine the probability that the observed correlation is statistically significant or not. A value of p < 0.05 was considered significant.

The study was conducted in accordance with the European Directive 2010/63/EU and the Italian Legislative Decree 26/2014. The study protocol was evaluated by the OPBA of the University of Camerino, which confirmed that the procedures did not constitute animal experimentation (Evaluation Code: OPBA13/2025). An informed consent form was signed by the owners, who were fully informed about the purpose and procedures of the study.

## 3. Results

**Figure 2:**
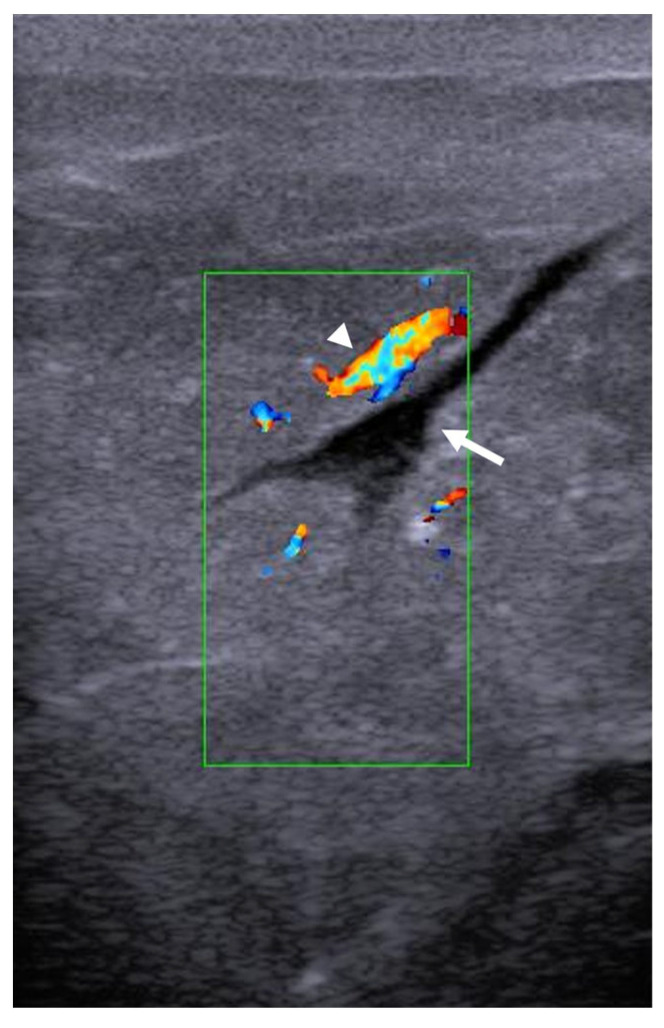
Sagittal ultrasonographic image of the udder parenchyma in a lactating jenny. The image demonstrates a homogeneous, coarsely granular, hypoechoic udder parenchyma with a visible lactiferous duct (white arrow). Color flow Doppler imaging aids in differentiating blood vessels (white arrowhead) from the lactiferous duct.

Ultrasonographic examination of the mammary parenchyma was easily performed in all jennies and revealed a uniform, finely hypoechoic echotexture with a coarse granular appearance, consistent with the normal architecture of active glandular tissue. Each mammary complex was composed of two distinct glandular units, each draining into a separate gland cistern. Within the parenchyma, lactiferous ducts appeared as elongated, branching anechoic tubular structures, lacking a defined echogenic wall. These ducts could be clearly distinguished from vascular structures, which also appeared as elongated, branching channels but were characterized by a thin, well-defined echogenic wall. The use of color flow Doppler imaging further facilitated differentiation, as vascular structures exhibited color-coded blood flow signals, whereas the lactiferous ducts remained anechoic and static, confirming their ductal rather than vascular nature (Figure 2).

Transcutaneous imaging of the teat structures was successfully achieved in all the lactating jennies. In longitudinal scans, teats appeared as cone-shaped and three-layered structure: an outer hyperechoic one corresponding to the skin, a thicker intermediate hypoechoic layer representing the muscular tissue, and a thin inner hyperechoic layer consistent with the mucosa. In all teats, both right and left, two distinct ductal systems were identified. Ultrasonographic evaluation of the teat structures allowed clear visualization of the teat canals, cisterns, and wall thickness, with all measurements easily obtained (Figure 3).

Specifically, the left teat exhibited a mean TCL of 1.85 ± 0.3 cm, TCD of 0.46 ± 0.2 cm, CrTWT of 0.58 ± 0.1 cm, and CaTWT of 0.75 ± 0.7 cm. Corresponding values for the right teat were 1.85 ± 0.3 cm, 0.38 ± 0.2 cm, 0.73 ± 0.9 cm and 0.82 ± 1.2 cm, respectively.

Descriptive statistics and correlations of the measurements are described in Table 1 and Table 2, in the corresponding order.

**Figure 4:**
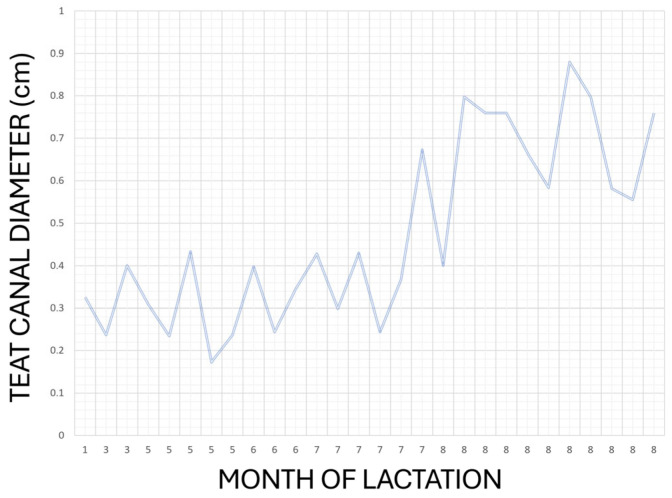
Graphical representation of relationship between month of lactation and TCD when left and right measurement are reported as single dataset.

Because the same operator performed both US scans acquisition and morphometric analysis and only few subjects for each age category were enrolled, Authors decided to limit statistical investigation only to descriptive statistics and correlations. As it is possible to observe in Table 1, no significant ultrasonographic changes were recorded between right and left teat for the considered variables. The Pearson correlation test highlighted a statistically significant correlation between TCD and the month of lactation in right and left teats (*p =* 0.040; *p =* 0.009, respectively; Table 2). Same result was obtained when data was cumulated in a single dataset for right and left side (*p =* 0.023) as can be possibly noticed in Figure 4.

No correlations were observed between the ultrasonographic measurements and age or body weight even when the data from both sides were cumulated into a single dataset.

## 4. Discussion

In farm animals, ultrasonographic examination of the mammary glands has been extensively studied. However, there are few reports describing the ultrasonographic anatomy of the teat structures in healthy lactating jennies. Knowledge of the normal anatomy in healthy individuals, along with the establishment of reference values, is essential for detecting early pathological changes, minimizing losses, and promoting animal welfare. The present study addresses the paucity of available information by providing detailed ultrasonographic measurements of dairy jennies’ teats, highlighting both anatomical features and adaptive changes throughout the lactation period.

In this context, particular attention was given to the ultrasonographic assessment of teat morphometric parameters, including TCL, TCD, and TWT. Considering the anatomical variability and the nonuniform diameter of the teat canal described in cattle [35], the present study adopted a standardized approach by measuring the TCD approximately at the midpoint between the teat orifice and the beginning of the teat cistern. This choice was made to minimize the potential bias related to proximal or distal canal variability and to ensure more consistent and comparable measurements among animals. In this study, teat wall thickness (TWT) was measured at the mid-height of teat canal. As previously reported, the Fürstenberg rosette in the mare is not clearly visualized and its presence remains uncertain [27]; therefore, teat canal length (TCL) was measured as the distance between the visible proximal end of the teat canal, at the transition to the teat cistern, and the distal end of the teat at the level of the teat orifice.

The results of the present study are in accordance with previous studies in equids [29,30] whose demonstrate no significant differences between left and right teat for all the measurements investigated, thus indicating a high degree of bilateral symmetry in teat anatomy among healthy jennies. This finding suggests a symmetrical functionality of the two glands, which may serve as a reliable reference for the early detection of unilateral pathological changes. Despite the small number of age-category-matched subjects, the ultrasonographic anatomy of the teats observed in this study is consistent with previous descriptions in mares and jennies [29,30], confirming a three-layered structure consisting of an outer hyperechoic layer corresponding to the skin, a thicker intermediate hypoechoic layer representing the muscular tissue, and a thin inner hyperechoic layer consistent with the mucosa.

We found no differences between age and weight in relation to the measurement. This could probably be due to low sample size, low variation in weight and unbalanced age group sizes. Further studies could better clarify this trend.

In the present study, it was not possible to make a fully reliable comparison with the results reported for lactating pony mares of similar size and weight [30], as the reference points and measurement procedures in that study were not described in sufficient detail. For example, the TWT was measured at the level of the teat cistern rather than at the teat canal, and TCL was taken from the structure referred to as the Furstenberg’s rosette, whose anatomical delineation was not clearly illustrated. Consequently, the exact proximal boundary of the TCL measurement cannot be confidently identified, which limits the strength of a direct comparison. Moreover, the authors assessed the teat cistern diameter rather than the teat canal diameter, as performed in the present study. Finally, the specific month of lactation in which their measurements were obtained was not reported, further reducing the accuracy and reliability of any potential comparison. Similarly, in previously published studies on jennies [29], the same methodological issues were observed. No clear anatomical reference points were defined for the TCL, TCD and TWT measurements, making it difficult to determine the exact location and extent of each parameter. Furthermore, the images suggest that their reported TCL included the length of the teat cistern. Similarly to Abshenas et al. [30], the TWT and TCD were measured at the level of the teat cistern and can therefore not be compared to the present study.

Moreover, the study by Hassan et al. [29] did not specify whether the ultrasonographic evaluations were performed before or after milking or suckling, a factor that may influence TWT measurements, as previously described by Stauffer et al. (2021) [36] for dairy cows.

It is therefore important that future studies, in addition to adopting a shared and standardized methodology, also provide detailed information on relevant variables such as the month of lactation, and whether the measurements were performed before or after milking or suckling, as well as the time interval from these events. Such details are essential to allow data harmonization of measurements and ensure an objective comparison of results across studies.

In the present study, TCD was highlighted to have a potential positive correlation with the month of lactation, progressively increasing throughout the lactation period. This can also be evaluated by observing the minimal and maximal values in Table 1, where a difference of about 6 mm is present between the 1st and the 8th months of lactation. Previous studies in dairy species have reported conflicting results regarding the relationship between TCD and lactation stage. In goats, a significant reduction in TCD was observed from the first to the fifth month of lactation, likely due to decreased milk volume and a progressively faster milk ejection [37]. In dairy cows, it has been demonstrated that TCD increases with the number of lactations, most likely as a consequence of mechanical exposure and traumatic effects associated with repeated milking [38]. Moreover, TCD has been shown to vary between healthy and mastitis-affected quarters, as reported by Klein et al. (2005) [38]. In 2016, Guarín and Ruegg [39] reported that a larger pre-milking teat apex diameter is associated with a higher risk of clinical mastitis, with a 20% increase in incidence for each 1 mm increase, underlining the clinical importance of canal morphology in bovine udder health. Similarly, Singh et al. (2017) [40] confirmed a positive correlation between TCD and somatic cell count, further supporting the role of teat canal morphology in mastitis susceptibility. Knowing the teat diameter during lactation is therefore crucial, as an enlarged teat canal can facilitate bacterial entry and significantly contribute to the onset of mastitis. While the previously cited studies associated larger teat canal diameter with higher somatic cell counts and mastitis risk, Seker et al. (2009) [41] reported contrasting results, showing that smaller ultrasonographic teat measurements were linked to CMT-positive cows. These discrepancies highlight the importance of further research to establish standardized ultrasonographic teat measurements in dairy animals, which could allow rapid identification of early signs of infection or inflammation and their risk factors.

A positive correlation between teat dimensions and milk yield has also been reported in camels [42], suggesting that increased teat diameter may represent a physiological adaptation to a greater milk storage capacity. Taken together with findings from other dairy species, these results indicate that variations in teat morphology can reflect both physiological adaptations and potential risk factors for disease, depending on the stage of lactation and species-specific anatomy. In donkeys, the progressive widening of the teat canal may similarly represent a species-specific physiological adaptation to lactational demands and milk flow dynamics; however, regardless of the underlying cause, an increase in teat canal diameter as lactation progresses may also be associated with a higher risk of mastitis.

Clinically, monitoring TCD during lactation could offer valuable insights into udder function and the early detection of pathological changes. Further studies are needed to validate this hypothesis and to clarify the potential role of ultrasonography in supporting udder health and preventing mammary gland diseases.

Prompt and accurate diagnosis of mammary gland disorders in farm animals is essential, as these conditions compromise animal welfare, reduce milk production, and increase costs for farmers [43]. As the first line of defense against intramammary infection, the teat canal prevents bacterial penetration and milk leakage through a smooth muscle sphincter at its distal orifice, which controls opening and closure [44].

Stenosis or obstruction of the teat canal, whether congenital or acquired, represents one of the most common teat pathologies in farm animals [45] and may result from inflammation, fibrosis, polyps, papillomas, milk stones, or traumatic lesions [43,45,46]. Although these conditions were not observed in the present study, probably due to the small number of enrolled animals, the ultrasonographic reference values herein provided may serve as a useful tool for clinicians to distinguish normal from pathological features during field examinations. However, these findings should be confirmed through larger, multicenter studies involving more jennies.

Besides obstructive lesions of the teat, mastitis has been associated with various changes across species, which can be detected both ultrasonographically and clinically. In dairy animals such as cattle and goats, both clinical and subclinical mastitis have been associated with ultrasonographic changes in teat morphology, including irregular outlines of the teat canal and cistern, hypoechogenic or hyperechoic contents, thickening of the teat wall, and loss of the normal three-layered wall structure [43,46,47]. Although the incidence of subclinical mastitis in equids is not well documented, it is likely more common than clinical mastitis, as observed in cows [48], highlighting the need for early and sensitive diagnostic approaches. Clinically, mastitis in the mare is often unilateral and, in some cases, may affect only one of the two ductal trees within the affected teat. It usually manifests with swelling, local heat, pain, purulent discharge, and ventral edema, with gland asymmetry and increased firmness also being common. In some cases, systemic signs and ipsilateral hindlimb lameness may accompany the inflammatory process [18,25]. Ultrasonography has proven useful in detecting chronic mastitis and mammary gland neoplasia, whereas acute mastitis often does not produce noticeable ultrasonographic changes [18,25]. Nevertheless, a complete evaluation of the teat and mammary gland may still be important to fully assess gland health. It should be noted, however, that in donkeys such evidence is lacking, and the usefulness of these ultrasonographic approaches in this species has not yet been established.

Despite the valuable aim of the study, several limitations may have reduced the strength of the results. First, the same operator performed both the US scans acquisition and morphometric analysis, and this may represent a bias in the measurements. It would have been ideal to have at least three different operators, blinded to the other findings, to compare and cross-validate their results; this aspect should be considered in future studies. It should be emphasized that the study was conducted under field conditions, without the aid of restraining devices or the controlled environment of a veterinary hospital and consequently with the difficulty of having a collaborative animal for a certain period. Therefore, the methodological approach was designed to balance accuracy and practicality, allowing the collection of potentially reliable and reproducible measurements without compromising the animal welfare or requiring invasive restraint, even if this accounted for the lack of different operators performing the same scan and anatomical analysis. Another limitation is the small number of animals included. This was due to a particular lack of collaboration of jennies’ owners; multicentred studies may attenuate this issue and may be more prone to obtain reliable data. However, unlike previous works that evaluated only early lactation stages [29,30], the present investigation covered a wider range of lactation months (1st to 8th) allowing a more comprehensive assessment of teat morphological changes. Future research should include larger populations of lactating jennies to confirm these findings and pathological cases can be included, as well as comparative evaluations with mares, to further explore the diagnostic potential of ultrasonography in identifying mammary gland alterations.

## 5. Conclusions

In summary, this study expands the baseline knowledge of the ultrasonographic anatomy of teat structures in healthy lactating jennies, an area that has been poorly explored to date. Transcutaneous ultrasonography proved to be a practical, rapid, and non-invasive diagnostic tool, feasible without physical or pharmacological restraint, and suitable for routine field assessments of udder health, even in rural areas with limited access to veterinary hospitals [49,50]. The standardized reference data presented here can support the development of advanced diagnostic protocols, ultimately improving milk quality, animal welfare and the economic sustainability of donkey farming, while providing a solid foundation for further research in this field.

## Figures and Tables

**Table 1:** Descriptive statistics of the measurements. Measures are expressed in cm.

	Variable	Mean (SD)	95% IC	Percentile				Min	Max
				10%	25%	75%	90%		
Right teat	TCL	1.85 (0.26)	1.84–1.86	1.54	1.67	2.01	2.05	1.20	2.51
	TCD	0.41 (0.19)	0.40–0.41	0.24	0.27	0.47	0.69	0.17	0.80
	CrTWT	0.73 (0.12)	0.70–0.76	0.45	0.50	0.63	0.70	0.31	0.89
	CaTWT	0.82 (0.11)	0.77–0.86	0.48	0.53	0.67	0.72	0.45	0.85
Left teat	TCL	1.85 (0.31)	1.84–1.86	1.51	1.68	1.94	2.26	1.41	2.66
	TCD	0.46 (0.16)	0.45–0.46	0.28	0.36	0.55	0.67	0.21	0.87
	CrTWT	0.58 (0.11)	0.58–0.58	0.47	0.48	0.67	0.71	0.39	0.85
	CaTWT	0.75 (0.73)	0.74–0.76	0.46	0.51	0.69	0.87	0.39	0.91

TCL = Teat canal length; TCD = Teat canal diameter; CrTWT = Cranial teat wall thickness; CaTWT = Caudal teat wall thickness.

**Table 2:** Association between measurements (mm) and age, body weight and month of lactation. r *=* correlation coefficient. Asterisk indicates significant correlation.

	Variable	Age		Weight		Month of Lactation	
		r	*p*-Value	r	*p*-Value	r	*p*-Value
Right mammary gland	TCL	0.045	0.428	−0.129	0.300	−0.231	0.171
	TCD	−0.361	0.064	−0.136	0.289	0.317	0.040 *
	CrTWT	0.092	0.355	−0.096	0.348	0.181	0.229
	CaTWT	0.102	0.339	−0.105	0.334	0.177	0.234
Left mammary gland	TCL	−0.101	0.340	−0.110	0.327	0.174	0.238
	TCD	0.005	0.493	−0.139	0.286	0.535	0.009 *
	CrTWT	0.346	0.073	0.190	0.218	0.304	0.103
	CaTWT	−0.137	0.288	0.014	0.478	0.078	0.375

TCL = Teat canal length; TCD = Teat canal diameter; CrTWT = Cranial teat wall thickness; CaTWT = Caudal teat wall thickness. The asterisk (*) indicates statistically significant values.

## Data Availability

The data presented in this study are available on request from the corresponding author. The data are not publicly available due to privacy restrictions.

## References

[1] Massouras T., Bitsi N., Paramithiotis S., Manolopoulou E., Drosinos E.H., Triantaphyllopoulos K.A. (2020). Microbial profile antibacterial properties and chemical composition of raw donkey milk. Animals.

[2] Claeys W.L., Verraes C., Cardoen S., De Block J., Huyghebaert A., Raes K., Dewettinck K., Herman L. (2014). Consumption of raw or heated milk from different species: An evaluation of the nutritional and potential health benefits. Food Control.

[3] Gastaldi D., Bertino E., Monti G., Baro C., Fabris C., Lezo A., Medana C., Baiocchi C., Mussap M., Galvano F. (2010). Donkey’s milk detailed lipid composition. Front. Biosci..

[4] Martemucci G., D’Alessandro A.G. (2012). Fat content, energy value and fatty acid profile of donkey milk during lactation and implications for human nutrition. Lipids Health Dis..

[5] Martini M., Altomonte I., Salari F., Caroli A.M. (2014). Monitoring nutritional quality of Amiata donkey milk: Effects of lactation and productive season. J. Dairy Sci..

[6] Salimei E., Fantuz F. (2012). Equid milk for human consumption. Int. Dairy J..

[7] Vincenzetti S., Pucciarelli S., Polzonetti V., Polidori P. (2017). Role of proteins and of some bioactive peptides on the nutritional quality of donkey milk and their impact on human health. Beverages.

[8] Vincenzetti S., Santini G., Polzonetti V., Pucciarelli S., Klimanova Y., Polidori P. (2021). Vitamins in human and donkey milk: Functional and nutritional role. Nutrients.

[9] Aspri M., Economou N., Papademas P. (2017). Donkey milk: An overview on functionality, technology, and future prospects. Food Rev. Int..

[10] Martini M., Altomonte I., Tricò D., Lapenta R., Salari F. (2021). Current knowledge on functionality and potential therapeutic uses of donkey milk. Animals.

[11] Laus F., Laghi L., Bazzano M., Cifone M.G., Cinque B., Yang Y., Marchegiani A. (2023). Donkey colostrum and milk: How dietary probiotics can affect metabolomic profile, alkaline sphingomyelinase and alkaline phosphatase activity. Metabolites.

[12] Li Y., Fan Y., Shaikh A.S., Wang Z., Wang D., Tan H. (2020). Dezhou donkey (*Equus asinus*) milk a potential treatment strategy for type 2 diabetes. J. Ethnopharmacol..

[13] Zhang Q., Sun W., Zheng M., Wang Q., Liu G., Li L., Zhang R., Zhang N. (2024). Donkey milk inhibits tumor growth by inducing apoptosis, pyroptosis and modulation of Th1/Th2 responses in a 4T1 murine breast cancer model. J. Funct. Foods.

[14] Khan M.Z., Chen W., Li M., Ren W., Huang B., Kou X., Qudrat U., Lin W., Tongtong W., Adnan K. (2024). Is there sufficient evidence to support the health benefits of including donkey milk in the diet?. Front. Nutr..

[15] Kazimierska K., Kalinowska-Lis U. (2021). Milk proteins—Their biological activities and use in cosmetics and dermatology. Molecules.

[16] Barbagianni M.S., Gouletsou P.G. (2023). Modern imaging techniques in the study and disease diagnosis of the mammary glands of animals. Vet. Sci..

[17] Tóth T., Tóth M.T., Abonyi-Tóth Z., Silva V., Poeta P., Sipos M., Juhász A. (2023). Ultrasound examination of the teat parameters of mastitis and healed udder quarters. Vet. Anim. Sci..

[18] Canisso I.F., Podico G., Ellerbrock R.E. (2021). Diagnosis and treatment of mastitis in mares. Equine Vet. Educ..

[19] Spiegel S., Spiegel F., Luepke M., Wendt M., von Altrock A. (2022). Ultrasonography and infrared thermography as a comparative diagnostic tool to clinical examination to determine udder health in sows. Animals.

[20] Dos Santos S.K., Oliveira M.G., Noriler E.P., Vrisman D.P., Borges L.P.B., Santos V.J.C., Coutinho L.N., Teixeira P.P.M. (2016). Mammary gland ultrasound evaluation of Jersey cattle breed. Acta Sci. Vet..

[21] Kotb E.E.Z., El-Fattah A., Ola A.A.E.F., Azab A.M., Leil A.Z. (2020). Ultrasonography, histopathological udder alterations and bacteriological investigations for diagnosis of mastitic goats. J. Appl. Vet. Sci..

[22] Abdullah O.M., Aslam S., Khan M.A., Mushtaq H., Hassan M., Akbar H., Hassan N., Ijaz M. (2023). Diagnosis and prognosis of bovine mastitis using ultrasonography and the associated risk factors on dairy farms. S. Afr. J. Anim. Sci..

[23] Hussein H.A., EL-Khabaz K.A., Malek S.S. (2015). Is udder ultrasonography a diagnostic tool for subclinical mastitis in sheep?. Small Rumin. Res..

[24] Martin L.M., Stöcker C., Sauerwein H., Büscher W., Müller U. (2018). Evaluation of inner teat morphology by using high-resolution ultrasound: Changes due to milking and establishment of measurement traits of the distal teat canal. J. Dairy Sci..

[25] Hughes K. (2021). Development and pathology of the equine mammary gland. J. Mammary Gland Biol. Neoplasia.

[26] McCue P.M. (2021). Evaluation of the mammary gland. Equine Reprod. Proced..

[27] Friker J., Ehlers J., Zeiler E., Liebich H.G. (2004). Ringfalten an der Stutenzitze. Pferdeheilkunde.

[28] D’Alessandro A.G., Mariano M., Martemucci G. (2015). Udder characteristics and effects of pulsation rate on milking machine efficiency in donkeys. J. Dairy Res..

[29] Hassan E.A., Abdelgalil A.I., Torad F.A., Shamaa A.A. (2016). Ultrasonographic examination of mammary glands in lactating Jennies (*Equus asinus*). Pak. Vet. J..

[30] Abshenas J., Sajjadian S.M., Taghavi M. (2014). Ultrasonographic examination of mammary glands in Caspian mares during the lactation and dry period. Iran. J. Vet. Surg..

[31] Crane M., Evans L. (2018). The Clinical Companion of the Donkey.

[32] Pilla R., Dapra V., Zecconi A., Piccinini R. (2010). Hygienic and health characteristics of donkey milk during a follow-up study. J. Dairy Res..

[33] Sarno E., Santoro A.M., Di Palo R., Costanzo N. (2012). Microbiological quality of raw donkey milk from Campania Region. Ital. J. Anim. Sci..

[34] Tavsanli H., Gökmen M., Önen A. (2020). Chemical and microbiological quality of donkey milk. Ank. Üniv. Vet. Fak. Derg..

[35] Wieland M., Melvin J.M., Virkler P.D., Nydam D.V., Heuwieser W. (2018). Development and evaluation of a standard operating procedure for ultrasound-based measurements of teat canal dimensions in dairy cows. J. Dairy Sci..

[36] Stauffer C., Van der Vekens E., Stoffel M.H., Schweizer D., Bruckmaier R.M. (2021). Increased teat wall thickness in response to machine milking. J. Dairy Sci..

[37] Fasulkov I., Karadaev M., Djabirova M. (2014). Ultrasound measurements of teat structures in goats. Rev. De Médecine Vétérinaire.

[38] Klein D., Flöck M., Khol J.L., Franz S., Stüger H.P., Baumgartner W. (2005). Ultrasonographic measurement of the bovine teat: Breed differences, and the significance of the measurements for udder health. J. Dairy Res..

[39] Guarín J.F., Ruegg P.L. (2016). Pre-and postmilking anatomical characteristics of teats and their associations with risk of clinical mastitis in dairy cows. J. Dairy Sci..

[40] Singh R.S., Bansal B.K., Gupta D.K. (2017). Relationship between teat morphological traits and subclinical mastitis in Frieswal dairy cows. Trop. Anim. Health Prod..

[41] Seker I., Risvanli A., Yuksel M.U.R.A.T., Saat N., Ozmen O. (2009). Relationship between California Mastitis Test score and ultrasonographic teat measurements in dairy cows. Aust. Vet. J..

[42] Atigui M., Brahmi M., Hammadi I., Marnet P.G., Hammadi M. (2021). Machine milkability of dromedary camels: Correlation between udder morphology and milk flow traits. Animals.

[43] Fasulkov I.R. (2012). Ultrasonography of the Mammary Gland in Ruminants: A Review. Bulg. J. Vet. Med..

[44] Berteau M., Pepler P.T., Broadhurst A., Hammond G., Zadoks R.N., Viora L. (2024). Assessing teat canal morphology in the dry period and during lactation by high-resolution ultrasound. J. Dairy Res..

[45] Ragab G.H., Seif M.M., Qutp M.M. (2016). Ultrasonography of the mammary gland in ruminants. J. Vet. Med. Res..

[46] Barbagianni M.S., Mavrogianni V.S., Vasileiou N.G.C., Fthenakis G.C., Petridis I.G. (2017). Ultrasonographic examination of the udder in sheep. Small Rumin. Res..

[47] Mourya A., Shukla P.C., Gupta D.K., Sharma R.K., Nayak A., Tiwari A., Singh B., Singh A.P., Sahi A., Jain A. (2020). Ultrasonographic alteration in subclinical mastitis in cows. J. Entomol. Zool. Stud..

[48] Perkins N.R., Threlfall W.R. (1993). Mastitis in the mare. Equine Vet. Educ..

[49] Schwarz T., Scheeres N., Małopolska M.M., Murawski M., Agustin T.D., Ahmadi B., Strzałkowska N., Rajtar P., Micek P., Bartlewski P.M. (2020). Associations between mammary gland echotexture and milk composition in cows. Animals.

[50] Accorroni L., Bazzano M., Marchegiani A., Spaterna A., Laus F. (2025). Abdominal Ultrasonography in Healthy Female Standard Donkeys. Animals.

